# Integrated DNA methylome and transcriptome analysis reveals the epigenetic regulatory mechanisms underlying maize response to copper stress

**DOI:** 10.1371/journal.pone.0329456

**Published:** 2025-08-28

**Authors:** Lin Zhao, Mengyan Zhang, Qi Wu, Xi Wu, Zhenyu Yun

**Affiliations:** 1 Sub-Institute of Agriculture and Food Standardization, China National Institute of Standardization, Beijing, China; 2 Agricultural Genomics Institute at Shenzhen, Chinese Academy of Agricultural Sciences, Shenzhen, China; China Three Gorges University, CHINA

## Abstract

DNA methylation plays a crucial role in plants’ adaptation to environmental stresses. However, the precise role of DNA methylation in regulating the response of maize (*Zea mays L.*) to copper stress remains incompletely understood. In this study, an integrated analysis of DNA methylome and transcriptome of the hybrid variety “Zhengdan 958” exposed to 1mM Cu stress at seedling stage was conducted using whole genome bisulfite sequencing (WGBS) and RNA-sequencing (RNA-seq). In the comparison between the control and copper stress sample, 3364 differentially expressed genes (DEGs) were detected (1637 upregulated and 1727 downregulated). The WGBS analysis revealed a genome-wide decrease in methylation levels across all cytosine contexts (CG, CHG, and CHH) under copper stress, with 1545 gene body hypomethylated differentially methylated genes (DMGs) and 1806 promoter hypomethylated DMGs. By integrating the analysis of DEGs and hypomethylated DMGs, we identified two predominant patterns of epigenetic regulation: (1) gene body CHG/CHH hypomethylation associated with transcriptional activation of metabolic and stress-response genes, and (2) promoter CHH hypomethylation linked to repression of developmental regulators and signaling components. This study provides valuable data for the epigenetic regulation of copper stress responses and identifies potential targets for developing stress-tolerant maize varieties through epigenetic breeding approaches.

## Introduction

Metals and metalloids with an atomic density exceeding 6 g cm^-3^ are classified as heavy metals [1]. Heavy metals contamination exhibits detrimental impacts on the growth and development of plants and crops [2,3]. Among heavy metals, copper (Cu) has garnered significant attention due to its dual properties to crops: essential at optimum levels and toxic at high levels [4,5]. Excessive copper is a prominent heavy metal pollutant, recognized for its potent toxicity and detrimental effects. Its tendency to accumulate in crops and persistently resist degradation significantly exacerbates the environmental concern [6,7]. Due to mining and smelting, industrial wastes and the use of agricultural chemicals containing copper, Cu toxicity has become a serious agricultural and environmental problem worldwide [8]. Cu toxicity can alter plant membrane permeability, interfere with the assimilation of essential nutrients and photosynthesis through reduction in pigment contents, cause oxidative stress, and influence root development and leaf expansion [9–11]. But the appropriate exposure to stress can also elicit a beneficial impact when encountering a secondary stress in crops [12].

Maize (*Zea mays L.*) is one of the most extensively cultivated cereal crops, with a total production surpassing that of wheat or rice [13]. Environmental risk and heavy metal-polluted waters can exert a negative effect on maize seedlings, causing yield and biomass reductions [14]. Exposure to excessive Cu triggers oxidative stress by accumulation of H_2_O_2_ in the maize leaves, and increased antioxidant enzymes activities to facilitate the restoration of cellular redox homeostasis [15,16]. RNA-Seq technology has been employed to examine gene expression patterns and identify stress-responsive genes involved in tolerance and stress response [17–19]. Therefore, performing transcriptome analysis may enrich the understanding of the Cu response mechanism of maize.

DNA methylation is a conserved epigenetic modification that plays a critical role in gene expression regulation, transposable element activity, genome stability and environmental adaptation [20,21]. In plants, DNA methylation can occur at cytosine residues in various sequence contexts, including CG, CHG (where H = A, T or C) and CHH [22,23]. Epigenome profiling in DNA methyltransferase (MTase) mutants of Arabidopsis revealed that MET1 (methyltransferase 1) maintains CG methylation, CMT3 (chromomethylase 3) maintains CHG methylation, while CHH methylation is preserved by DRM1/2 (domains rearranged methyltransferases 1 and 2) and CMT2 (chromomethylase 2) [24]. Increased numbers of studies have shown that alteration of DNA methylation at individual gene loci or across the entire genome contribute to tolerance and adaptation to abiotic stresses in plants [25,26]. For instance, cadmium stress altered the expression of key genes involved in cytosine methylation regulation in kenaf [27]. DNA methylation in the promoter of *ZmNAC111* can repress gene expression, resulting in increased drought sensitivity in maize seedlings [28].

With the development and deployment of omics approaches using high-throughput processing, genome-wide analyses of methylation patterns enable the detection of differentially methylated regions and the quantification of methylation at each specific gene position [29]. A comprehensive analysis of DNA methylation profiles in maize roots has revealed that 140 differentially methylated genes are potentially implicated in response to Pb stress [30]. Moreover, DNA methylation enhances rice tolerance to heavy metal stress by mitigating its toxic effects. Notably, Cong et al. [31] demonstrated that DNA hypomethylation-mediated transcriptional reprogramming contributes to heavy metal (mercury) resistance in rice. However, the understanding of epigenetic mechanisms that underlie maize’s response to copper stress remains limited and incomplete. In this study, whole genome DNA methylation combined with transcriptome gene expression analyses was conducted in the control and Cu treatment maize seedlings roots to elucidate the molecular mechanisms governing the response of maize to copper stress. Through the integration of DNA methylome and transcriptome, this work could provide a better understanding of the copper-tolerance mechanism in maize.

## Materials and methods

### Culture and treatment of plant material

Maize seeds from the hybrid line “Zhengdan 958” were first sterilized and imbibed in distilled water for 12 h. Subsequently, the imbibed seeds were germinated and the resulting seedlings were cultivated in a growth cabinet maintained at 25 °C, with 70% humidity, a 16-hour light cycle followed by an 8-hour dark period. For the study on Cu stress, Copper sulfate (CuSO_4_·5H_2_O) was utilized. The 7-day-old seedlings were grown in Petri dishes, with half of them being subjected to a 1mM Cu treatment, while the other half served as a control without any Cu treatment. After one week, the control and Cu treatment seedlings roots were collected for analysis. Each sample was independently repeated three times (CK and Cu represented the control and Cu treatment seedlings, respectively).

### Transcriptome sequencing

Total RNA was extracted from each root sample by following the instructions provided with the Trizol reagent kit (Invitrogen, Carlsbad, CA, USA). RNA integrity was evaluated using the RNA Nano 6000 Assay Kit on the Bioanalyzer 2100 system (Agilent Technologies, CA, USA). The cDNA libraries were constructed and sequenced on an Illumina Novaseq platform (Illumina, San Diego, CA, USA) by Novogene Co., LTD. (Beijing, China).

To obtain high-quality clean reads, the raw reads were filtered by removing those that contained adapters, reads with poly-N and low-quality reads. An index of the reference genome was constructed and the paired-end clean reads were aligned to the maize B73 reference genome using Hisat2 v2.0.5. [32]. The mapped reads were assembled by StringTie (v1.3.3b) [33].

Gene expression levels were calculated using the fragments per kilobase million (FPKM) method [34]. Differential expression analysis between groups (three biological replicates per group) was performed using the DESeq2 Bioconductor package (1.20.0). The P-values obtained from the analysis were adjusted using the Benjamini and Hochberg method to control the false discovery rate. Genes that exhibited fold change ≥2 and adjusted P-value ≤0.05 were considered to be differentially expressed. GO enrichment analysis of DEGs was conducted using the GOseq R package, which corrected for gene length bias. GO terms with corrected P-value ≤0.05 were defined as significant enrichment. The statistical enrichment of differentially expression genes in the Kyoto Encyclopedia of Genes and Genomes (KEGG) pathways was conducted using the clusterProfiler R package.

### WGBS

The total genomic DNA was extracted from each root sample using the Hi-DNAsecure Plant Kit (TIANGEN, Beijing, China). The extracted DNA was then fragmented into 200–400-bp using Covaris S220 system (Covaris, USA). Bisulfite conversion was performed with the EZ DNA Methylation-Gold Kit (Zymo Research, USA), with denaturation at 94°C for 5 min and conversion at 64°C for 2.5 h, while spiking in λ-DNA as an internal control for conversion efficiency. Subsequently, methylation sequencing adapters ligation, size selection and PCR amplification were performed on the DNA fragments. Using the Accel-NGS Methyl-Seq DNA Library Kit (Swift, USA), BS-seq libraries were prepared. The quality of these libraries was evaluated using the Agilent 5400 system (Agilent Technologies, CA, USA). Finally, pair-end sequencing of the sample was carried out on an Illumina platform (Illumina, San Diego, CA, USA).

The raw data obtained from sequencing underwent quality control analysis using FastQC (fastqc_v0.11.8). For data analysis, briefly, Bismark (v0.24.0) was used to perform alignments of bisulfite-treated reads to the latest maize B73 reference genome (B73 RefGen_v5). To identify differentially methylated regions (DMRs), the DSS software was employed [35]. Gene Ontology (GO) enrichment analysis for DMR-related genes was conducted using the GOseq R package. Additionally, KEGG enrichment analysis for genes related to DMRs was performed using the KOBAS software.

### Quantitative real-time PCR analysis

Total RNA was converted into cDNA using a reverse transcription kit provided by TaKaRa (Tokyo, Japan). Quantitative real-time PCR was carried out with SYBRPremix Ex TaqTM II (also from TaKaRa) and monitored by a Bio-Rad CFX Connect Real-time system (Bio-Rad, CA, USA). The PCR cycle was conducted using a three-step method, and the relative expression was calculated using the 2^─∆∆CT^ method. GAPDH served as the reference gene and the primers used are listed in S1 Table. Each sample was subjected to three biological replicates.

## Results and discussion

### RNA sequencing and transcriptomic analysis

To gain a comprehensively understanding of the impact of copper stress on the gene expression profile of maize seedlings, the total RNA extracted from their root tissues was sequenced using the Illumine platform. A Cu concentration of 1000 μmol/L was found to inhibit the root growth (S1 Fig) [6]. The experiment included six samples in total, with CK (control) and Cu (1mM Cu stress) groups, each consisting of three biologic replicates. RNA sequencing resulted in from 40.96 million to 43.32 million raw reads for each root sample. The clean reads generated were between 39.11 million and 41.27 million. The Q20 and Q30 values were consistently above 97.03% and 92.10%, respectively (Table 1). These parameters suggested that the quality of sequencing data is sufficient for subsequent transcriptome analyses. Principal Component Analysis (PCA) of CK and Cu samples was performed to identify the overall variance in the transcript data. The PCA results indicate that the gene expression in CK and Cu groups were obviously distinguished, the first and second principal component accounted for 60.62% and 16.47% of variance, respectively (S2 Fig).

**Table 1:** Statistics of RNA-seq for control and Cu-treated samples of maize seedlings.

sample	raw_reads	clean_reads	Mapping rate(%)	Unique Mapped rate(%)	Q20	Q30	GC_pct
CK1	44635186	43510628	87.81	85.60	97.38	92.82	54.67
CK2	42952478	41949454	87.53	85.42	97.26	92.47	54.82
CK3	43985048	43274122	85.55	83.40	97.09	92.10	54.17
Cu1	43344676	41007624	88.22	86.18	97.17	92.43	56.12
Cu2	44175272	42693824	88.75	86.78	97.31	92.75	56.67
Cu3	45423512	42782062	87.92	85.89	97.03	92.16	56.05

**Fig 1:**
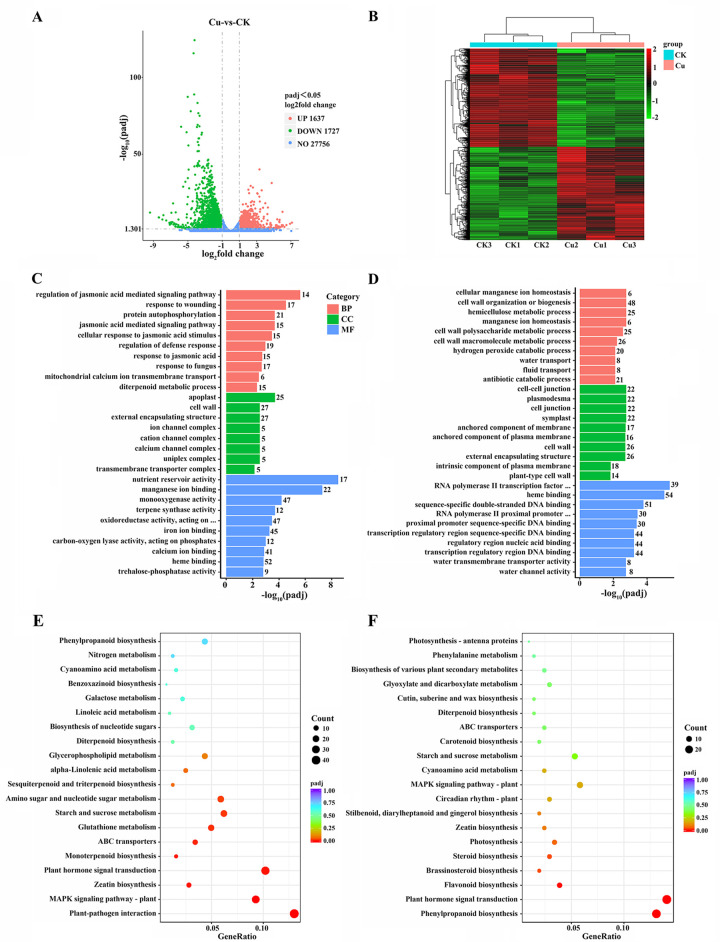
Transcriptomic profiling of maize seedlings in response to copper stress. **(A)** Volcano plot of all detected genes; Upregulated genes are represented by red dots, down-regulated genes by green dots, and genes with no transcriptional change by blue dots. **(B)** Expression heatmap clustering based on all DEGs; Darker red indicates higher expression, while darker green indicates lower expression. GO enrichment analysis of the down-regulated genes (**C**) and up-regulated genes (**D**) detected in the Cu vs. CK comparison. The color red corresponds to biological process (BP), green to cellular component (CC), and blue to molecular function (MF). KEGG pathway enrichment analysis of the down-regulated genes (**E**) and up-regulated genes (**F**) detected in the Cu vs. CK comparison. The circle size represents number of genes, circle color represents padj. Gene ratio is the ratio of the enriched genes to the total number of genes in the relative pathway. P-values were adjusted by the Benjamini-Hochberg method.

Transcriptomics analysis of maize seedlings from both control and copper stress conditions were conducted to investigate the molecular changes that occur in response to copper stress. The transcriptome analysis revealed 3364 DEGs between control and copper-stressed maize seedlings, with 1637 genes upregulated and 1727 genes downregulated (Fig 1A). Heatmap of hierarchical clustering analysis revealed that these DEGs effectively distinguished between CK samples and Cu samples (Fig 1B), indicating distinct transcriptional responses to copper stress.

The transcriptomic analysis revealed distinct patterns between down- and up-regulated genes. The GO terms of down-regulated genes related to cell wall, external encapsulating structure, apoplast, heme binding, monooxygenase activity, oxidoreductase activity and iron ion binding were significantly enriched (Fig 1C). The GO terms of up-regulated genes were related to transcription factor and heme binding (Fig 1D). KEGG metabolic pathways for down-regulated genes, including plant hormone signal transduction, MAPK signaling pathway, phenylpropanoid biosynthesis and plant-pathogen interaction were notably enriched (Fig 1E). Conversely, up-regulated genes demonstrated activation of metabolic and defensive systems, including enhanced phenylpropanoid/flavonoid biosynthesis for structural and antioxidant production, improved hormone signaling capacity, and elevated photosynthetic activity (Fig 1F). These changes indicate a strategic shift toward metabolite production and environmental adaptation under the experimental conditions.

These coordinated changes in defense, metabolism and signaling pathways reflect a complex physiological reprogramming under experimental conditions. The integrated GO and KEGG analyses not only elucidate the plant’s adaptive strategies but also identify key candidate pathways for future functional studies on plant stress adaptation and survival mechanisms.

### Genome-wide patterns of DNA methylation and comparative methylation analysis

Epigenetic modifications, particularly DNA methylation, play a pivotal role in regulating gene expression and shaping the plant’s adaptive responses to stress [36–38]. DNA methylation variations are widespread in maize and can contribute significantly to phenotypic variation [39]. To explore the regulatory mechanisms of methylation levels in maize under copper stress, we conducted WGBS analysis. A total of 286.94 million and 349.36 million raw reads were generated for the control and Cu-treatment samples. Following the removal of low-quality data, 280.37 million clean reads from the control group and 340.33 million clean reads from the copper-treated group were successfully aligned to the reference genome. The mapping rates were 75.34% and 75.41%, respectively. The conversion efficiency of unmethylated cytosine (C) to thymine (T) was very high, exceeding 99% for all the libraries used in the analysis (Table 2). These data demonstrate that the methods and results had high reliability and accuracy.

**Table 2:** Statistics of WGBS for control and Cu-treated samples of maize seedlings.

Sample	Raw Reads	Clean Reads	Mapped reads	Q20(%)	Q30(%)	Mapping rate(%)	BS Conversion Rate(%)	Unique Mapped rate(%)
CK	300879143	293988105	221483641	97.23	91.65	75.34	99.566	46.05
Cu	366331634	356858845	269101557	96.87	90.9	75.41	99.661	48.20

**Fig 2:**
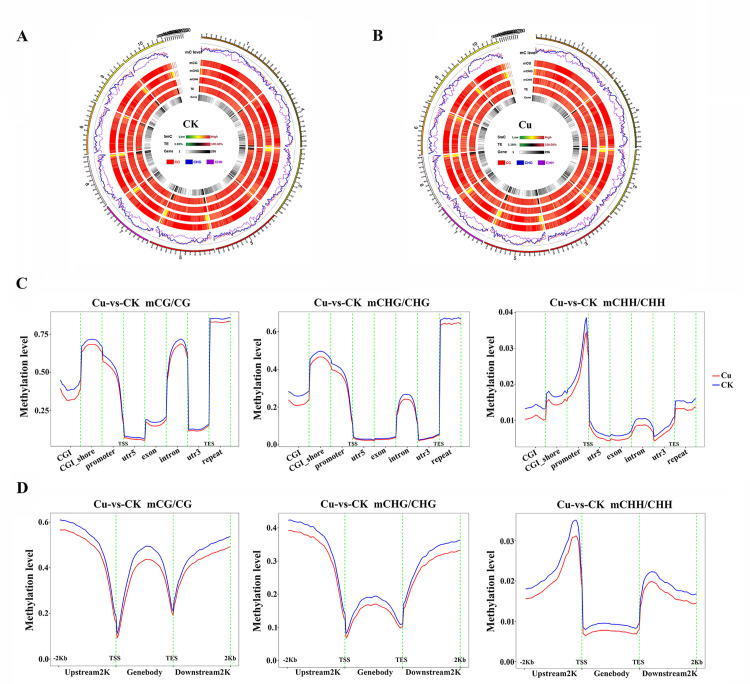
Genome-wide DNA methylation patterns and distribution in control and copper-treated groups. A circle plot of DNA methylation density, gene density, transposon density, and DNA methylation levels across 10 chromosomes of control (**A**) and copper-treated (**B**) groups. From the outside to the inside, mC level indicates the methylated cytosine in the CG (red), CHG (blue), and CHH (purple) contexts; mCG, mCHG, and mCHH indicate the methylated cytosine density in each context; TE indicates transposon density; Gene indicates gene density, respectively. **(C)** The comparison of methylation levels on gene functional elements in the CG, CHG, and CHH contexts. The x-axis shows genomic elements, and the y-axis displays methylation levels. Each gene region was split into 20 bins, with mean C-site methylation levels calculated per bin. **(D)** The comparison of methylation levels on the upstream 2K, gene body, and downstream 2K in the CG, CHG, and CHH contexts. The x-axis represents distinct genomic regions, while the y-axis denotes methylation levels. Gene regions were split into 50 bins, with mean C-site methylation calculated per bin. Colors represent experimental groups.

In the control (CK) samples, the proportion of methylated cytosine at CG, CHG and CHH sites relative to the total methylated cytosine sites were 50.29%, 42.57% and 7.14%, respectively (S3A Fig). When subjected to copper stress, there was a slight change in the methylated cytosines proportions in three contexts, with mCG and mCHG decreased to 49.88% and 42.11%, mCHH increased to 8.00% (S3B Fig). Global DNA methylation profiles of chromosomes 1–10 are shown in Fig 2A and B. A circos map was used to visualize the methylation density and level across the chromosomes of both the CK and Cu groups, and the results revealed a potential positive correlation between DNA methylation and transposable element (TE) density, as well as a negative correlation between DNA methylation and gene density. These findings align with previous studies indicating that environmental cues can trigger epigenetic modifications, which in turn regulate gene expression patterns in plants [40,41].

The DNA methylome analysis provides insights into the epigenetic modifications that occur in response to environmental stressors, revealing how these modifications can influence gene expression without altering the genetic code [42]. Understanding these changes is crucial for comprehending the long-term adaptive responses of plants to environmental challenges. Specifically, we perform a genome-wide methylation profiling analysis to compare the methylation levels of various genomic functional regions between the CK and Cu groups. For each cytosine context (CG, CHG, CHH), we performed statistical analyses to determine the average methylation levels of cytosine sites within various genomic functional regions, including promoter, exon, intron, CpG islands (CGI), CGI shore, repeat, and others (Fig 2C). Copper treatment significantly decreased CG and CHH methylation in all examined regions, whereas CHG methylation reduction in 5’UTR, exon and 3’UTR regions was not significant. Additionally, we examined the 2K-bp region upstream of the transcription start site (TSS), the gene body, and the 2K-bp region downstream of the transcription termination site (TTS) (Fig 2D). Significantly, CG context exhibited higher methylation levels in genomic functional regions, and DNA methylation levels showed decreased trends during Cu treatment in all CG, CHG, and CHH contexts. This observation suggests that copper stress induces a genome-wide demethylation effect in maize, which may be a part of the plant’s response mechanism to cope with the stress. And these results emphasize the significance of DNA methylation as a rapid and adaptable epigenetic mark that can modulate gene expression and maintain genome stability in response to copper stress.

### DMRs enrichment analysis

**Fig 3:**
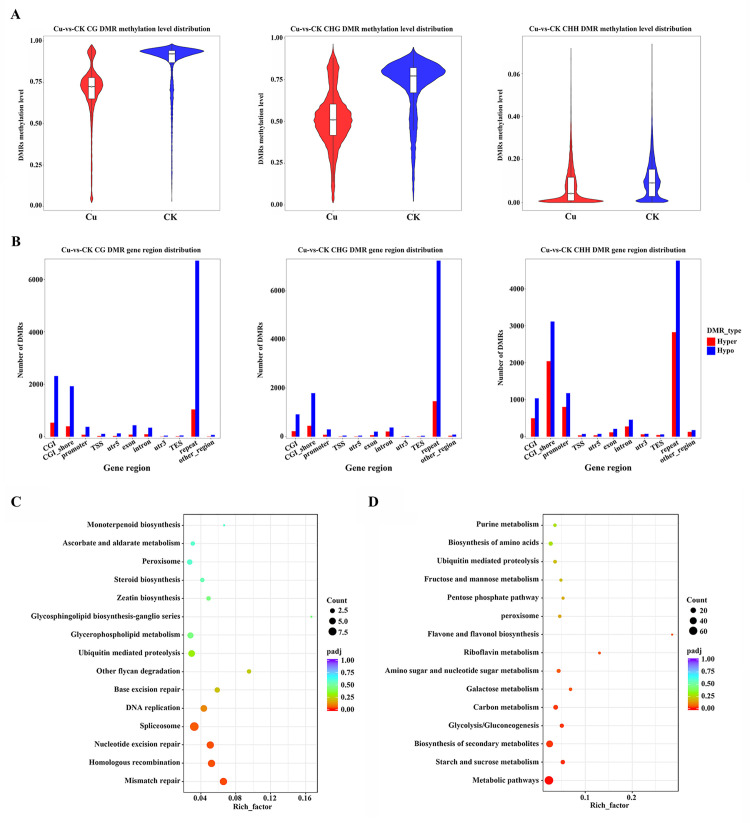
Genome-wide analysis of differential DNA methylation and functional enrichment in copper-treated versus control groups. **(A)** Methylation level distribution of DMRs in the CG, CHG, and CHH contexts by violin boxplots. Colors represent experimental groups. **(B)** Number of DMRs in the CG, CHG, and CHH contexts in different regions across the genome. Hypermethylated DMRs are shown in red, while hypomethylated DMRs are indicated in blue. Cu-vs-CK, copper-treated group versus control group. KEGG pathway enrichment of hypomethlyated DMGs in the CHG **(C)**, and CHH (**D**) contexts. The circle size represents number of genes, circle color represents padj. The Rich Factor represents the proportion of differentially expressed genes relative to all genes annotated in a pathway. P-values were adjusted by the Benjamini-Hochberg method.

Based on WGBS analysis, we observed notable changes in DNA methylation patterns in maize under copper stress. One of the key aspects of DNA methylation research is the identification of DMRs between different samples or conditions. Following exposure to copper stress, a reduction in the overall methylation level of DMRs was detected in all CG, CHG, and CHH contexts (Fig 3A). DMRs effectively distinguished between CK samples and Cu samples according to the heatmap of hierarchical clustering (S4 Fig). Further detailed comparative analysis showed that hypermethylated DMRs or hypomethylated DMRs were primarily distributed in CGI, CGI_shore, promoter, exon, intron and repeat regions. Notably, the number of hypomethylated DMRs in these regions was significantly increased under cooper stress, especially for CG and CHG contexts (Fig 3B). The observed hypomethylation in promoter regions may lead to altered expression of stress-responsive genes. Similarly, changes in methylation patterns within exons, introns, and repeats could affect gene splicing, transcript stability, or the regulation of non-coding RNAs, all of which could contribute to the plant’s stress response.

Furthermore, a total of 1545 gene body hypomethylated DMGs and 1806 promoter hypomethylated DMGs were identified through the gene annotation of DMRs. Kyoto KEGG enrichment analysis was performed to determine the biological functions of these genes. Hypomethylated DMGs in the CHG context were involved in mismatch repair, homologous recombination and nucleotide excision repair (Fig 3C). And the top three pathways of hypomethylated DMGs in the CHH context were metabolic pathways, starch and sucrose metabolism and biosynthesis of secondary metabolites (Fig 3D). The results of KEGG enrichment revealed that DNA methylation patterns in the CHG context may significantly impact the maintenance of genome stability and the DNA repair machinery, and hypomethylation in the CHH context could have profound effects on primary and secondary metabolic processes, potentially influencing the organism’s overall metabolism and its ability to adapt to environmental changes.

### Association between DNA methylome and transcriptome

**Fig 4:**
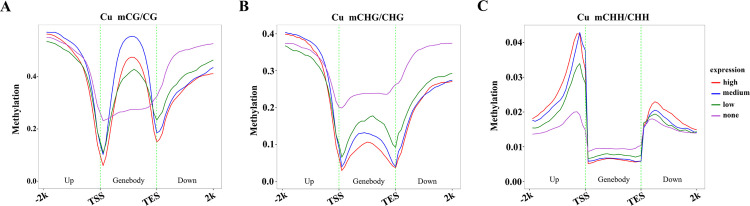
Correlation analysis between gene expression and DNA methylation for the CG (A), CHG (B), and CHH (C) contexts. The expressed genes were equally divided into four groups according to the expression value from none to high. Gene regions were split into 50 bins, with mean C-site methylation calculated per bin.

The combined analysis of DNA methylome and transcriptome is particularly potent as it allows for the direct correlation of epigenetic modifications with gene expression changes. This integrated approach can uncover the regulatory networks and molecular mechanisms underlying stress tolerance [43]. To evaluate the correlation between gene expression level and methylation level, all genes were proportionally divided into none, low, medium or high, according to their expression levels, and the average methylation level was calculated for each of these groups. The findings revealed that the CG methylation level in the gene body region of medium group was higher than that observed in the other groups (Fig 4A), which is in accordance with previous studies in other plant species [44,45]. Conversely, genes characterized by the highest expression levels (categorized as the ‘high’ group) displayed reduced CHG and CHH methylation levels in gene body (Fig 4B and C). Gene body methylation exhibited negative correlations with expression levels in both CHG (Spearman’s rho ρ: −0.46; *p* < 0.01) and CHH (Spearman’s rho ρ:-0.26; *p* < 0.01) context. This inverse relationship between CHG and CHH methylation levels and gene expression suggests a potential suppressive role of these methylation marks in transcriptional activity. Such findings are consistent with the hypothesis that non-CG methylation, particularly CHG and CHH, may serve as a regulatory mechanism to repress transcription, thereby contributing to the epigenetic regulation of gene expression [46,47]. CHH methylation levels in the upstream 2kb regions appear to be positively correlated with expression levels (Spearman’s rho ρ:0.27; *p* < 0.01), which is consistent with known findings that lower CHH methylation levels frequently occur at promoters of genes with decreased expression in rice and maize [48,49]. Collectively, these findings demonstrate the complexity of DNA methylation-mediated gene regulation, highlighting the context-specific roles of different methylation marks in modulating transcriptional output.

### Integrated analysis of DEGs and hypomethylated DMGs

**Fig 5:**
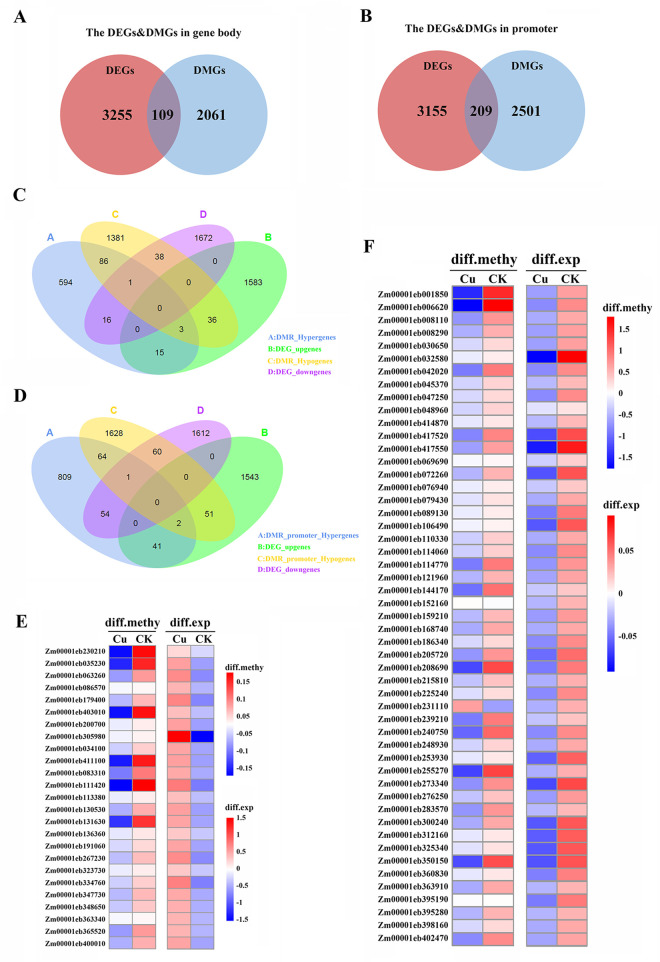
Integrated analysis of DMGs and DEGs. **(A)** Venn diagram of DEGs and DMGs in gene body. **(B)** Venn diagram of DEGs and DMGs in promoter. **(C)** Venn diagrams showing DMGs (Hyper/Hypo in gene body) and DEGs (up/down-regulated) for each comparison. **(D)** Venn diagrams showing DMGs (Hyper/Hypo in promoter) and DEGs (up/down-regulated) for each comparison. DMR_Hypergenes, genes anchored by DMRs with hypermethylation levels in the gene body region; DMR_Hypogenes, genes anchored by DMRs with hypomethylation levels in the gene body region; DMR_promoter_Hypergenes, genes anchored by DMRs with hypermethylation levels in the promoter region; DMR_promoter_Hypogenes, genes anchored by DMRs with hypomethylation levels in the promoter region; DEG_upgenes, up-regulated genes in transcriptomic expression; DEG_downgenes, down-regulated genes in transcriptomic expression. **(E)** Identifies the association of gene body hypomethylation (CHG/CHH) with the upregulated expression of 25 candidate genes. **(F)** Identifies the association of promoter hypomethylation (CHH) with the downregulated expression of 51 candidate genes.

Our integrated multi-omics analysis reveals a complex epigenetic regulatory network underlying maize responses to copper stress, characterized by distinct methylation-expression relationships in different genomic regions (Fig 5A–5D). The study identified two predominant patterns of epigenetic regulation: (1) gene body CHG/CHH hypomethylation associated with transcriptional activation of metabolic and stress-response genes, aligns with previous studies indicating that gene body methylation is negatively correlated with gene expression levels in plants [50,51], and (2) promoter CHH hypomethylation linked to repression of developmental regulators and signaling components.

Our integrated analysis identified 25 candidate genes exhibiting coordinated gene body CHG/CHH hypomethylation and transcriptional upregulation (Table 3 and Fig 5E), revealing a distinct epigenetic regulatory pattern compared to promoter-associated methylation changes. These genes encode functionally diverse proteins, including metabolic enzymes (e.g., *AAE1*, *PAR1*, *AGL2*), transcription factors (e.g., *MYB36*), metal transporters (e.g., *NRAM4*, *LSI3*), and cell wall modifying enzymes (e.g., *CSLF2*, *XYXT1*). Notably, several stress-responsive genes (e.g., *R13L3*, *PER66*) and signaling components (e.g., *P2C66*, *PERK7*, *ROGF7*) were also identified, suggesting potential roles in adaptive responses.

**Table 3:** 25 candidate genes that exhibited both up-regulated in expression and CHG/CHH hypomethylation status in the gene body region.

gene_id	UniProt ID	gene_name	C_context	gene_description
Zm00001eb230210	F4HUK6	*AAE1*	CHG	Butanoate-CoA ligase AAE1
Zm00001eb035230	Q6Z8P4	*PIRL4*	CHG	Plant intracellular Ras-group-related LRR protein 4
Zm00001eb063260	A0A0B6VQ48	*PAR1*	CHG	Phenylacetaldehyde reductase
Zm00001eb086570	Q9FKL2	*MYB36*	CHG	Transcription factor MYB36
Zm00001eb179400	P25776	*ORYA*	CHG	Oryzain alpha chain
Zm00001eb403010	PF05340	*/*	CHG	Protein of unknown function (DUF740)
Zm00001eb200700	Q9STE7	*R13L3*	CHG/CHH	Putative disease resistance RPP13-like protein 3
Zm00001eb305980	Q9F234	*AGL2*	CHG/CHH	Alpha-glucosidase 2
Zm00001eb034100	Q6ZKL8	*P2C66*	CHH	Probable protein phosphatase 2C 66
Zm00001eb411100	Q9LQ32	*umc2705*	CHH	Glucuronoxylan 4-O-methyltransferase 3
Zm00001eb083310	P59226	*H32*	CHH	Histone H3.2
Zm00001eb111420	A5H454	*PER66*	CHH	Peroxidase 66
Zm00001eb113380	Q5QN13	*NRAM4*	CHH	Metal transporter Nramp4
Zm00001eb130530	Q8VY22	*TBL38*	CHH	Protein trichome birefringence-like 38
Zm00001eb131630	B4FAP1	*CSPL4*	CHH	CASP-like protein 1E1
Zm00001eb136360	Q9C6A1	*MTEFE*	CHH	Transcription termination factor MTERF15
Zm00001eb191060	Q9AV23	*LSI3*	CHH	Silicon efflux transporter LSI3
Zm00001eb267230	Q9FGG3	*WTR45*	CHH	WAT1-related protein At5g64700
Zm00001eb323730	Q84S11	*CSLF2*	CHH	Mixed-linked glucan synthase 2
Zm00001eb334760	Q5Z8T8	*XYXT1*	CHH	Beta-1,2-xylosyltransferase XYXT1
Zm00001eb347730	Q9LZN0	*ROGF7*	CHH	Rop guanine nucleotide exchange factor 7
Zm00001eb348650	Q94CC0	*Y5994*	CHH	Uncharacterized protein At5g49945
Zm00001eb363340	/	/	CHH	/
Zm00001eb365520	M4IQQ7	*CCL9*	CHH	Probable CoA ligase CCL9
Zm00001eb400010	Q9XI96	*PERK7*	CHH	Proline-rich receptor-like protein kinase PERK7

**Fig 6:**
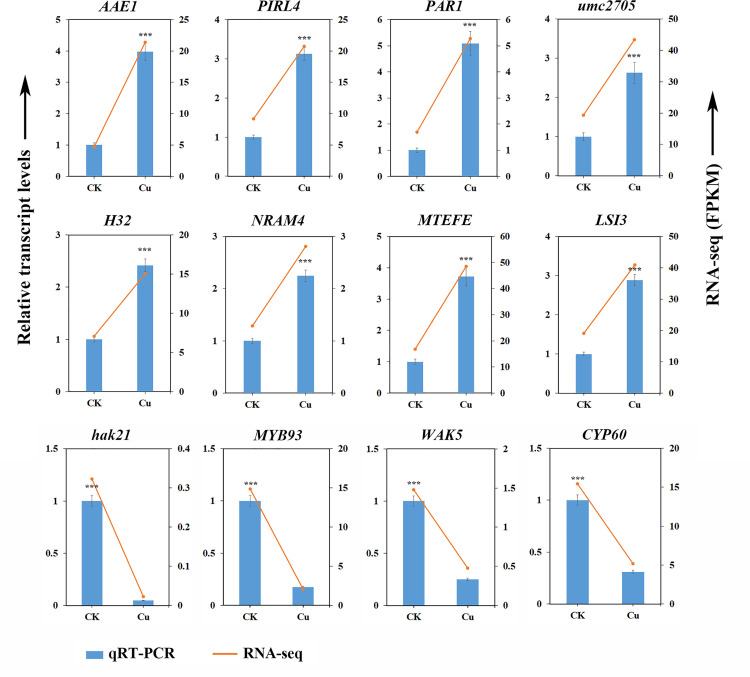
Validation of the expression levels of response genes under copper stress by real-time quantitative PCR. Bars represented means±SD from three biological replicates. Triple asterisks represented *p* < 0.001 in comparison to control by Student’s *t*-*t*est.

Conversely, the promoter hypomethylation pattern involved 51 genes encoding transcription factors (e.g., *WRKY19*, *MYB93*, *GRAS49*, *WRKY78*, *HB41*), receptor kinases (e.g., *SIT2*, *SD18*, *WAK5*, *CRK23*), and enzymes involved in metabolic processes (e.g., *BCH2*, *SAT4*, *GAUT6*, *ME5*, *NCED5*) (Table 4 and Fig 5F). Notably, the repression of stress-responsive genes (e.g., *TLP*, *BI1*, *SIP1*) through this mechanism suggests a potential role in environmental adaptation. The expression patterns of these genes exhibited a high degree of consistency with the results obtained from transcriptome sequencing (Fig 6). The positive relationship between promoter CHH hypomethylation and expression implies that loss of methylation in this context may contribute to transcriptional repression, possibly through indirect mechanisms such as altered chromatin accessibility or recruitment of repressive complexes.

**Table 4:** 51 candidate genes that exhibited both down-regulated in expression and CHH hypomethylation status in the promoter region.

gene_id	UniProt ID	gene_name	C_context	gene_description
Zm00001eb001850	O49814	*BCH2*	CHH	Beta-carotene hydroxylase 2
Zm00001eb006620	Q9SE96	*GEML1*	CHH	GEM-like protein 1
Zm00001eb008110	Q10QH1	*SAT4*	CHH	Probable serine acetyltransferase 4
Zm00001eb008290	PF00168	*/*	CHH	C2 domain
Zm00001eb030650	O48716	*JGB*	CHH	Protein JINGUBANG
Zm00001eb032580	P31110	*TLP*	CHH	Thaumatin-like protein
Zm00001eb042020	Q0DFT7	*WRK19*	CHH	WRKY-transcription factor 19
Zm00001eb045370	/	*/*	CHH	/
Zm00001eb047250	Q7XUN6	*SIT2*	CHH	L-type lectin-domain containing receptor kinase
Zm00001eb048960	Q75G84	*hak21*	CHH	Potassium high-affinity transporter21
Zm00001eb414870	P46573	*PBL10*	CHH	Probable serine/threonine-protein kinase
Zm00001eb417520	K7U185	*/*	CHH	/
Zm00001eb417550	C0HIJ6	*LTI6B*	CHH	Hydrophobic protein
Zm00001eb069690	O81905	*SD18*	CHH	Receptor-like serine/threonine-protein kinase
Zm00001eb072260	Q9S9Z2	*MYB93*	CHH	MYB-related-transcription factor 93
Zm00001eb076940	Q9LMN7	*WAK5*	CHH	Wall-associated receptor kinase 5
Zm00001eb079430	A0A1D6E8B6	*aga5*	CHH	Alkaline galactosidase5
Zm00001eb089130	A0A0N9HT29	*C71BE*	CHH	Desmethyl-deoxy-podophyllotoxin synthase
Zm00001eb106490	A0A1D6EXP9	*gras49*	CHH	GRAS-transcription factor 49
Zm00001eb110330	A0A1D6F2F4	*Zm00001eb110330*	CHH	helical bundle-like domain
Zm00001eb114060	Q9LMN86Q2	*WAK3*	CHH	Wall-associated receptor kinase 3
Zm00001eb114770	Q9M9Y5	*GAUT6*	CHH	Probable galacturonosyltransferase 6
Zm00001eb121960	A0A1D6MIP2	*/*	CHH	/
Zm00001eb144170	Q5N712	*JM705*	CHH	Lysine-specific demethylase
Zm00001eb152160	B6SZY7	*gst8*	CHH	Glutathione transferase8
Zm00001eb159210	A0A1D6NI45	*cyp60*	CHH	Cytochrome P450 60
Zm00001eb168740	A0A1D6PS58	*/*	CHH	/
Zm00001eb186340	K7UHN0	*nrg10*	CHH	Nitrate regulatory gene10
Zm00001eb205720	O48788	*Y2267*	CHH	Probable inactive receptor kinase
Zm00001eb208690	Q9MBD8	*BI1*	CHH	Bax inhibitor 1
Zm00001eb215810	Q54TC5	*NTPPB*	CHH	7-methyl-GTP pyrophosphatase
Zm00001eb225240	Q03730	*YMB8*	CHH	Uncharacterized vacuolar membrane protein
Zm00001eb231110	Q9SCK1	*LSU1*	CHH	Protein RESPONSE TO LOW SULFUR 1
Zm00001eb239210	C0LGG9	*Zm00001d016242*	CHH	Probable LRR receptor-like serine/threonine-protein kinase
Zm00001eb240750	A0A1D6H7X4	*wrky78*	CHH	WRKY-transcription factor 78
Zm00001eb248930	A0A1D6HER1	*hb41*	CHH	Homeobox-transcription factor 41
Zm00001eb253930	/	*/*	CHH	/
Zm00001eb255270	A0A1D6HKQ3	*/*	CHH	/
Zm00001eb273340	O65482	*CRK23*	CHH	Putative cysteine-rich receptor-like protein kinase 23
Zm00001eb276250	A7NY33	*PER4*	CHH	Peroxidase 4
Zm00001eb283570	B6TVG1	*me5*	CHH	Malic enzyme5
Zm00001eb300240	B6SV18	*nced5*	CHH	Nine-cis-epoxycarotenoid dioxygenase5
Zm00001eb312160	A0A1D6I407	*myb159*	CHH	MYB-transcription factor 159
Zm00001eb325340	P33679	*sip1*	CHH	Stress-induced protein1
Zm00001eb350150	Q9FJF5	*SOK5*	CHH	Protein SOSEKI 5
Zm00001eb360830	PF08263	*/*	CHH	Leucine rich repeat N-terminal domain
Zm00001eb363910	Q9ZVM9	*Y1461*	CHH	Probable serine/threonine-protein kinase
Zm00001eb395190	PF11331	*/*	CHH	Probable zinc-ribbon domain
Zm00001eb395280	Q9ZVI7	*GAUT7*	CHH	Probable galacturonosyltransferase 7
Zm00001eb398160	A0A1D6PEA2	*mkkk10*	CHH	MAP kinase kinase kinase10
Zm00001eb402470	A0A1D6PJD1	*ms25*	CHH	Male sterile25

These findings significantly advance our understanding of context-dependent DNA methylation functions in plants, demonstrating how spatial patterns of methylation and sequence context collectively shape transcriptional responses to environmental stress. The identification of these epigenetic signatures provides both mechanistic insights into copper stress adaptation and potential molecular markers for breeding stress-resilient maize varieties through epigenetic selection strategies.

## Conclusions

The integrated analysis of the DNA methylome and transcriptome of maize seedlings exposed to excess copper stress provides new insights into the molecular mechanisms governing the stress response. The transcriptomic analysis revealed 3364 differentially expressed genes, of which 1637 were upregulated and 1727 downregulated, indicating a complex transcriptional reprogramming in response to copper stress. The DNA methylome analysis showed a global decrease in methylation levels across all cytosine contexts (CG, CHG, and CHH) under copper stress, with 1545 hypomethylated gene body DMGs and 1806 promoter hypomethylated DMGs identified. By integrating the results from both analyses, we identified 25 genes exhibiting gene body CHG/CHH hypomethylation and transcriptional upregulation, 51 genes exhibiting promoter CHH hypomethylation and transcriptional downregulation. The observed association between gene body hypomethylation and transcriptional activation of stress-responsive and metabolic genes suggests an adaptive mechanism to enhance copper tolerance. Conversely, promoter hypomethylation-mediated repression of growth-related genes likely reflects resource reallocation to prioritize stress defense. These findings demonstrate the context-dependent roles of DNA methylation, where gene body hypomethylation facilitates transcriptional activation of stress-adaptive pathways, while promoter hypomethylation mediates repression of growth-related genes. The study provides new insights into the epigenetic regulation of copper stress responses and identifies potential targets for developing stress-tolerant maize varieties through epigenetic breeding approaches. Future studies should further explore the functional significance of these differentially expressed and hypomethylated genes in maize stress adaptation and their potential roles in crop improvement.

## Supporting information

S1 Fig Phenotypic analysis of maize seedlings under copper stress. (**A**) Root growth status of maize seedlings treated with 1mM Cu. (**B**) Root growth status of maize seedlings treated with 1mM Cu. (**C**) Root weight of maize seedlings treated with 1mM Cu.
(TIF)

S2 Fig Correlation analysis among samples. (**A**) Pearson correlations among samples. A Pearson correlation coefficient closer to 1 indicates a greater similarity in expression patterns among samples. (**B**) PCA plot of samples of maize seedlings grown under control and copper stress conditions. CK_1, CK_2, and CK_3 represent samples from control seedlings, while Cu1, Cu2, and Cu3 represent the samples from seedlings exposed to copper stress.
(TIF)

S3 Fig Proportional distribution map of methylated cytosines. Distribution of three contexts (CG, CHG, and CHH) of methylated cytosines for the control (**A**) and copper-treated (**B**) group. Different colors represent methylated cytosines in different contexts, and the size of each part area represents the proportion of methylated cytosines in the corresponding context.
(TIF)

S4 Fig The clustering heatmap of DMRs methylation level for the CG, CHG, and CHH contexts. Cu-vs-CK, copper-treated group versus control group.
(TIF)

S1 Table Primer sequences for qRT-PCR. (DOCX)
