# Supplementary figures and images for: Integrated DNA methylome and transcriptome analysis reveals the epigenetic regulatory mechanisms underlying maize response to copper stress
Source: PLoS One. 2025 Aug 28;20(8):e0329456. doi: 10.1371/journal.pone.0329456 (PMC12393742; doi:10.1371/journal.pone.0329456)

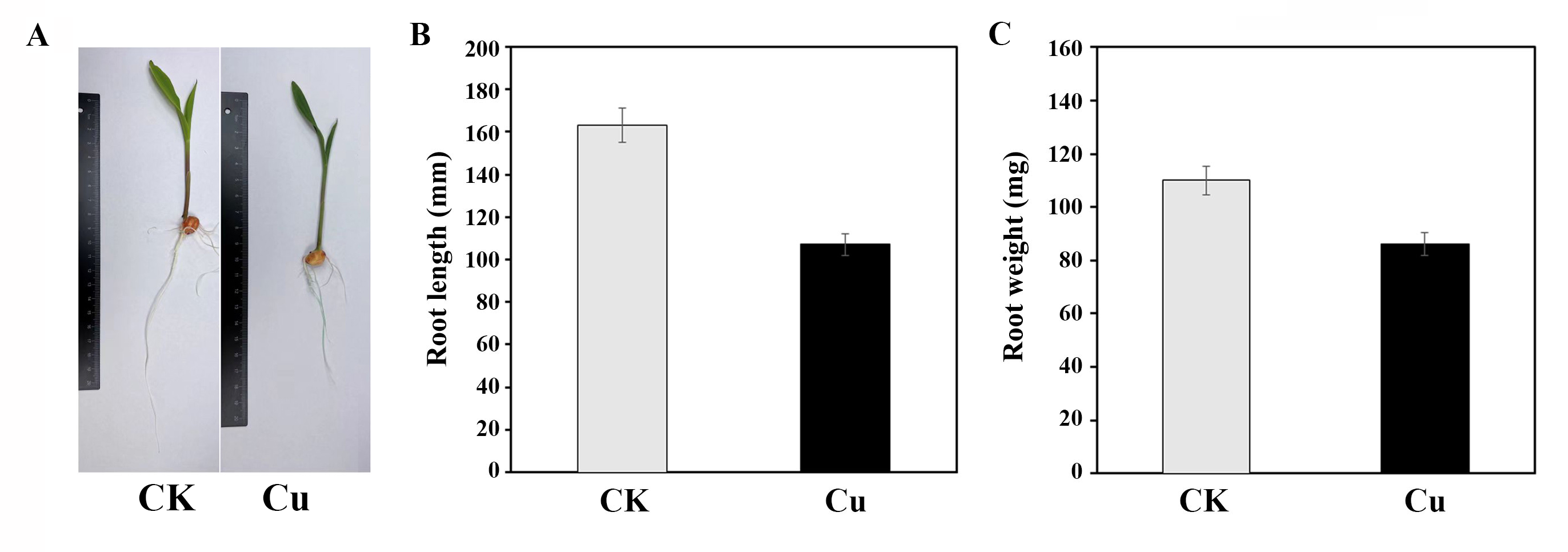

Supplement: S1 Fig — (A) Root growth status of maize seedlings treated with 1mM Cu. (B) Root growth status of maize seedlings treated with 1mM Cu. (C) Root weight of maize seedlings treated with 1mM Cu. (TIF) [file pone.0329456.s001.tif]

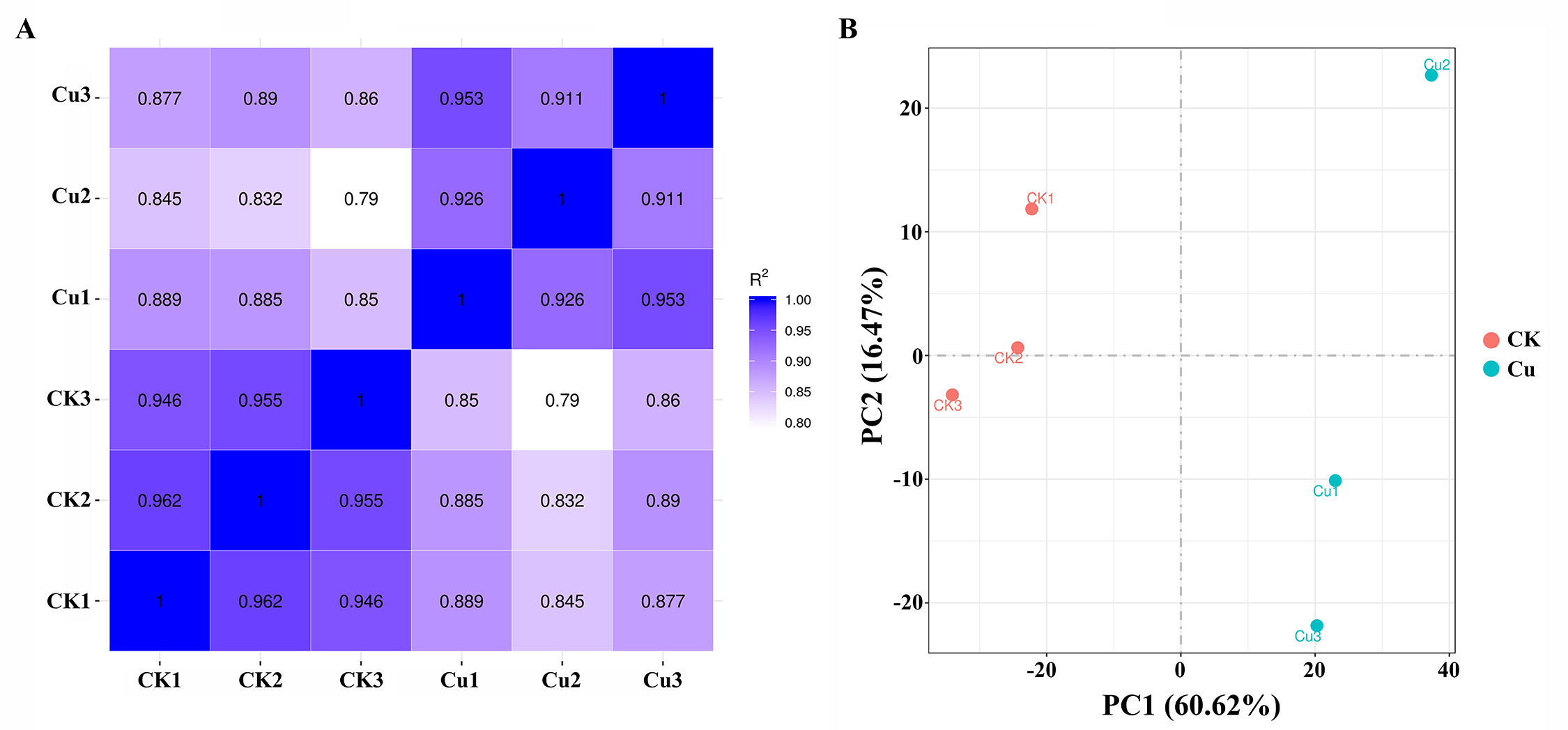

Supplement: S2 Fig — (A) Pearson correlations among samples. A Pearson correlation coefficient closer to 1 indicates a greater similarity in expression patterns among samples. (B) PCA plot of samples of maize seedlings grown under control and copper stress conditions. CK_1, CK_2, and CK_3 represent samples from control seedlings, while Cu1, Cu2, and Cu3 represent the samples from seedlings exposed to copper stress. (TIF) [file pone.0329456.s002.tif]

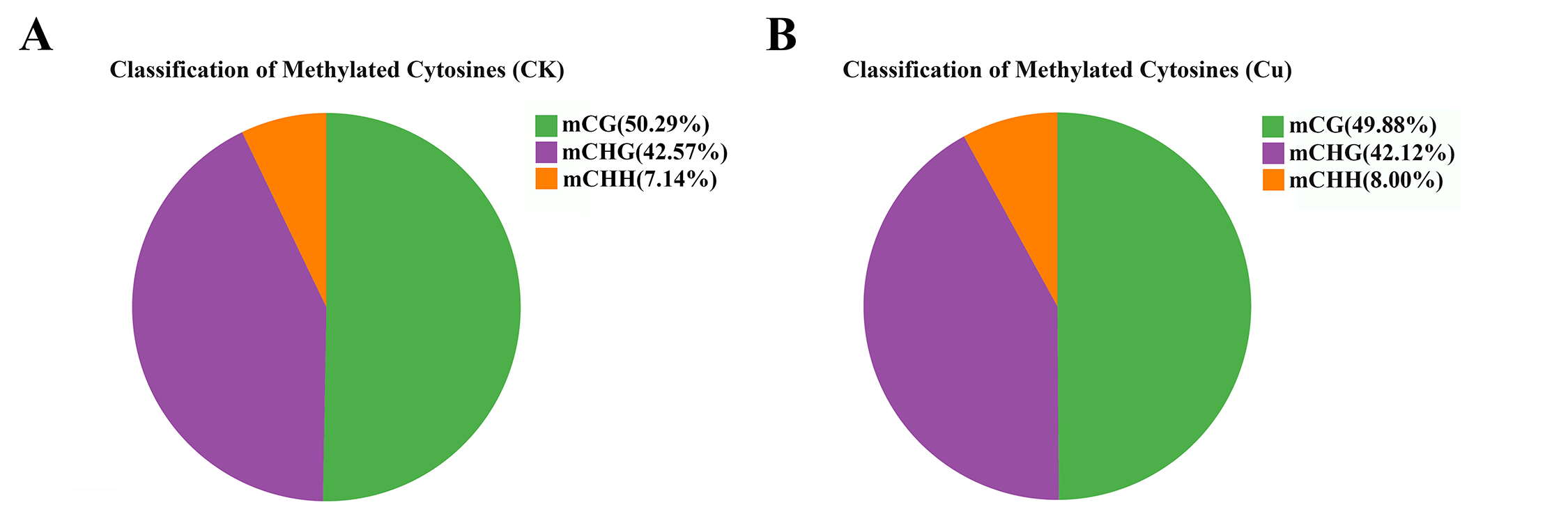

Supplement: S3 Fig — Distribution of three contexts (CG, CHG, and CHH) of methylated cytosines for the control (A) and copper-treated (B) group. Different colors represent methylated cytosines in different contexts, and the size of each part area represents the proportion of methylated cytosines in the corresponding context. (TIF) [file pone.0329456.s003.tif]

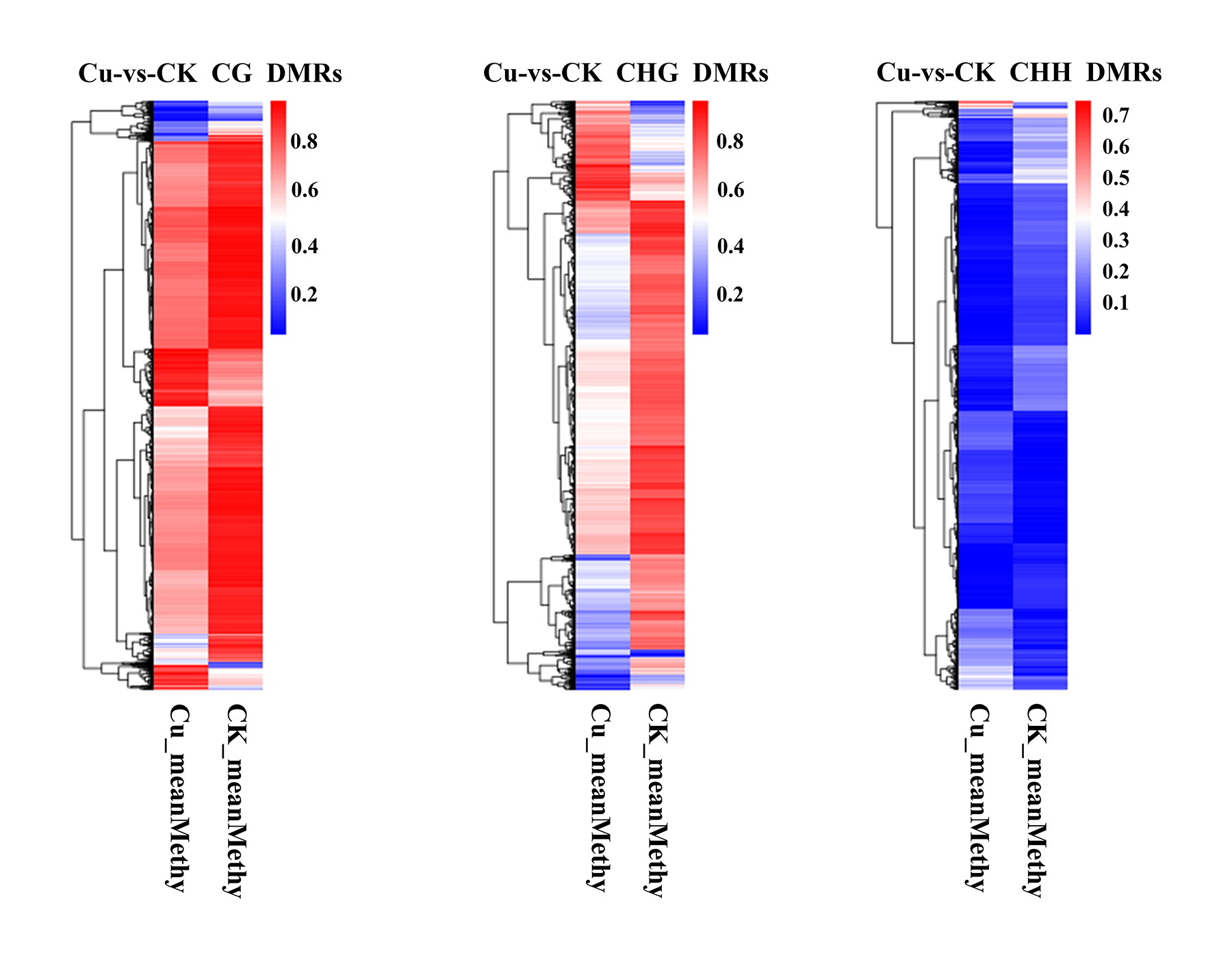

Supplement: S4 Fig — Cu-vs-CK, copper-treated group versus control group. (TIF) [file pone.0329456.s004.tif]
