# Supplementary material for: Integrated DNA methylome and transcriptome analysis reveals the epigenetic regulatory mechanisms underlying maize response to copper stress
Source: PLoS One. 2025 Aug 28;20(8):e0329456. doi: 10.1371/journal.pone.0329456 (PMC12393742; doi:10.1371/journal.pone.0329456)
Supplement: S1 Table — (DOCX) [file pone.0329456.s005.docx]

Table S1 Primer sequences for qRT-PCR

| Gene | Sequence (5′-3′) |
| --- | --- |
| *AAE1*  (Zm00001eb230210) | CTACTCGGGCAAATCGAGAG  ACGTGGTAAGTCTCTGTCCA |
| *PIRL4*  (Zm00001eb035230) | TCTGTGGCGAGTTTTGTTCA  GTGTCTATTCGGTCGTGCTT |
| *PAR1*  (Zm00001eb063260) | TCCGACGAACTGAAGGTGAT  ACAACAGCCCTAGTCGAGTC |
| *umc2705*  (Zm00001eb411100) | CCATGTGCCCAGGAGAAAAT  GTTTGTTTGGATCATGGCGG |
| *H32*  (Zm00001eb083310) | CGTCCATATCCATCCAACGG  CGTAGAGGAGTGAGCAGTCT |
| *NRAM4*  (Zm00001eb113380) | CTGAACCAGAAGCTTGATTACA  CGGCGTTGCGACCTTAG |
| *MTEFE* (Zm00001eb136360) | AAGGCAACAGTGGCTGTAAA  AGCTCTGAATACTGCAAGCC |
| *LSI3* (Zm00001eb191060) | TCGGGTTAATCAGAGCCAGA  AGTGTTGCTATAACGCGAGA |
| *hak21*  (Zm00001eb048960) | CGCTTGGTGACATCCTCCTT  GCAAAATGTTCGGGGTGCTT |
| *MYB93*  (Zm00001eb072260) | CGGACGGACAACGAGATCAA  CGCTTCTTCCTCCGTCGTTA |
| *WAK5*  (Zm00001eb076940) | GTGGCGGCCTTCTATTCCTT  AATATCTTCGTGCCGTCGCT |
| *Cyp60*  (Zm00001eb159210) | CCTCCCAGGTTTCAGGTTCC  CTGGTCTCAGCGATGTTGGA |
| *GAPDH* | CTGGTGCTGGAATTGCACTG  TCAATGACGCGGTTGCTGTA |
